# Unveiling the Hidden Connection between Allergies and Sleep-Disordered Breathing in Children and Its Impact on Health

**DOI:** 10.3390/children10071143

**Published:** 2023-06-30

**Authors:** Marco Zaffanello, Giorgio Piacentini

**Affiliations:** Department of Surgery, Dentistry, Paediatrics and Gynaecology, University of Verona, 37126 Verona, Italy

Sleep-disordered breathing (SDB) and allergies in children are increasingly relevant health issues that require attention. SDB encompasses various conditions, including obstructive sleep apnea (OSA), snoring, and mouth breathing, all of which impede normal airflow during sleep. As a result, children with SDB may be more easily awoken and disrupted during sleep and more likely to experience a reduction in overall sleep quality [1]. On the other hand, allergies involve an abnormal immune response to common substances such as pollen, dust mites, and animal dander, resulting in symptoms like itchy eyes, a runny nose, sneezing, and general fatigue [2]. SDB and allergies can significantly affect quality of life in children, impacting their physical, emotional, and cognitive well-being [3].

Research on the association between SDB and allergies has yielded substantial evidence highlighting the connections between these disorders [4]. Specifically, studies have focused on pediatric populations. OSA has been linked to the development of allergies such as allergic rhinitis, asthma, and eczema, as well as the severity of allergic rhinitis and OSA itself [5]. Notably, the severity of OSA has been found to correlate with the frequency of asthmatic symptoms. Investigations have also explored the relationship between snoring frequency and asthma symptoms [6]. Additionally, the impact of pollen exposure on the exacerbation of SDB has been considered [7]. Polysomnography (PSG) has been examined to gain better insights into the connection between SDB and allergies, providing implications for treatment, early detection, and timely intervention [4]. Lastly, research has also delved into allergists’ perceptions and practices regarding SDB and allergies [8].

Evidence demonstrates a correlation between the severity of allergic rhinitis and the severity of OSA [9]. Persistent and moderate/severe allergic rhinitis may increase the risk of developing OSA [7,10]. Likewise, OSA has been associated with the suboptimal control of asthma and more severe chronic asthma symptoms [11]. Habitual snoring has been associated with heightened asthma symptoms in children, poorer asthma control, and increased healthcare utilization [6]. Respiratory allergies can also lead to nasal inflammation, congestion, and upper airway obstruction, increasing the likelihood of SDB [7].

Assessing SDB and allergies requires various tools and approaches. Several assessment methods have been suggested, including the use of the Pediatric Sleep Survey Instrument to evaluate sleep-related problems among children [12]. The Strengths and Difficulties Questionnaire assesses the psychological distress associated with SDB. Evaluating allergic disease frequency can help identify the conditions that worsen SDB [12]. Polysomnography (PSG) is a valuable tool used to record various physiological variables during sleep and can help evaluate the presence of snoring and the severity of SDB [13]. The Allergic Rhinitis and its Impact on Asthma (ARIA) classification system is used to assess the impact of allergic rhinitis on quality of life, and medical history, clinical examinations, and allergen testing can provide detailed information about an individual’s medical background and any specific allergies they may have [10]. Lastly, pollen counting during PSG enables the evaluation of the influence of allergens on sleep quality [7].

The management of SDB and allergies necessitates a multifaceted and personalized approach [8]. When multiple disorders have been identified in an individual, it is crucial to use an integrated approach that not only considers the interactions between the individual and their conditions but also could facilitate the establishment of a comprehensive treatment plan. Other essential aspects of the management of SDB and allergies include utilizing appropriate pharmacological therapies and customizing the treatment based on the patient’s clinical and personal characteristics [7,8,14]. It is also necessary to consider the presence of comorbidities in asthma control and allergy management [11]. The appropriate use of medication, identification of risk factors, and specialized medical consultation play a vital role in effectively managing these conditions [3].

Research on SDB and allergies has shed light on various areas of study and led to significant findings. Associations between allergic diseases, sleep problems, and psychological distress have been identified, providing insights into the interconnectedness of these conditions. Studies have demonstrated a correlation between the apnea–hypopnea index and the severity of allergic rhinitis, indicating a relationship between SDB and allergic respiratory disease [10,15]. Additionally, an association has been found between OSA and the severity of acute asthma, highlighting the impact of SDB on asthma outcomes. Snoring has also been linked to increased asthma morbidity, emphasizing the connection between sleep disturbances and respiratory health [6].

Ongoing research is primarily focused on exploring allergic rhinitis, the long-term effects of respiratory allergy, and developing effective therapies for SDB [5]. Efforts are being made to establish personalized management strategies based on the latest scientific evidence to improve the care and well-being of individuals affected by these conditions. These research endeavors are being conducted to enhance our understanding of the complex relationship between SDB and allergies and will hopefully provide valuable insights for developing targeted interventions and treatments.

Table 1 contains a comprehensive analysis of the relationship between SDB and allergies, specifically allergic rhinitis and asthma; highlights the interplay between these conditions, including their associations, management strategies, and potential implications for future research and medical practice; contains valuable insights into the connections between SDB and allergies; and presents data on the association between SDB and allergic rhinitis, demonstrating how these conditions often coexist and affect each other. Moreover, the table delves into the interaction between SDB, allergic rhinitis, and asthma, illustrating how these conditions can exacerbate one another and create a more challenging clinical scenario while highlighting the importance of a multidisciplinary approach to managing these interconnected conditions effectively.

Research on SDB and allergies has provided valuable insights and raised several key points with significant future implications. In managing allergic diseases in children, it is crucial to consider sleep issues and incorporate long-term asthma monitoring to ensure the provision optimal care [5,16].

Further research into the relationship between eczema and SDB is necessary, along with investigations into the connections between allergies, sleep disturbances, and psychological distress [12]. Both the assessment and management of allergic rhinitis in children with OSA are paramount. More studies are needed to enhance our understanding of the interplay between OSA, allergic rhinitis, and SDB, which can significantly affect management strategies. Additionally, considering pollen counting and accurately assessing OSA in patients with acute asthma are vital considerations for effective treatment [7]. Allergists play a proactive role in diagnosing and managing SDB, and their involvement is crucial in providing comprehensive care [8].

Identifying effective preventive and therapeutic strategies, adopting improved screening protocols, and detecting symptoms and underlying causes are essential future goals. A comprehensive understanding of the interactions between SDB and allergies can pave the way for developing integrated clinical guidelines, facilitating targeted and comprehensive management approaches. Addressing these conditions requires a collaborative effort among researchers, clinicians, and policymakers to improve quality of life among children affected by SDB and allergies. Through collaboration, we can develop evidence-based interventions and policies that prioritize the well-being of these individuals and provide them with the support they need.

In conclusion, SDB and allergies are significant health concerns that mutually influence each other and can significantly influence quality of life among children. Extensive research has shed light on the association between SDB and the development of allergies, including allergic rhinitis, asthma, and eczema. Moreover, the severity of SDB has been found to affect the severity of allergies. In isolation, allergies can increase susceptibility to SDB by triggering inflammation in the upper respiratory tract, while SDB can disrupt sleep patterns and exacerbate allergy symptoms. Evaluating and managing these conditions requires specific tools and approaches, such as PSG and testing, to assess sleep patterns and identify specific allergens.

A personalized and multidisciplinary approach is crucial for effectively managing SDB and allergies in children. This includes tailoring treatment plans to individual patients, employing appropriate pharmacological therapies, and seeking specialized medical consultations when necessary. Despite notable advancements in research, some areas still require further investigation. These areas for improvement include bettering our general understanding of the interactions between SDB, allergies, and psychological distress; detecting symptoms early; and developing preventive and therapeutic strategies. By comprehensively understanding these connections, integrated clinical guidelines can be established, improving children’s well-being and outcomes for those affected by SDB and allergies.

## Figures and Tables

**Table 1:** An analysis of the association between sleep-disordered breathing and allergies, particularly allergic rhinitis and asthma and their evaluation, management, and future implications for medical research and practices.

Association	Evaluation	Management	Future Implications
Association between the severity of allergic rhinitis and the severity of OSA.	Evaluated via the completion of questionnaires on quality of life in relation to allergic rhinitis and ARIA classification [10].	Management of allergic rhinitis.	Assessing and managing allergic rhinitis in children with OSA.
Prevalence of significant allergic rhinitis among children with SDB [4].	Assessed by reviewing one’s medical history, clinical examinations, and allergy tests.	Management of allergic rhinitis.	Accurate evaluation and proper management of allergic rhinitis in children with SDB.
Association between the severity of OSA and the severity of allergic rhinitis [10].	Polysomnography.	Management of OSA.	Long-term monitoring and management of OSA in children with allergic rhinitis.
OSA is associated with suboptimal disease control and increased asthma severity [5].	Evaluation of hospitalized patients with acute asthma and the presence of OSA.	Integrated management of OSA and acute asthma.	Accurate assessment of OSA in patients with acute asthma for better disease control.
Association between OSA and increased risk/duration of hospitalization found in both obese and non-obese patient groups [5].	Evaluation of clinical outcomes based on the presence of obesity.	Integrated management of OSA and acute asthma in obese and non-obese patients.	Specific OSA assessments and the management of both obese and non-obese patients.
Association between children hospitalized with OSA and acute asthma [5].	Evaluation of clinical outcomes in children.	Integrated management of OSA and acute asthma management in children.	Assessment and management of OSA in children with acute asthma to improve clinical outcomes.
Children in urban areas are particularly affected by asthma and SDB [14].	Assessment of snoring frequency and asthma symptoms.	Integrated SDB and asthma management.	Assessment of snoring in children with asthma for better disease control.
Habitual snoring is associated with an increase in asthma symptoms and greater use of healthcare facilities than those who do not snore [6].	Assessment of asthma symptoms and frequency of snoring.	Integrated SDB and asthma management.	Accurate assessment of snoring frequency to identify patients at risk of worsening asthma.
Habitual snoring is associated with worse asthma control [6].	Asthma control assessment based on the presence of chronic snoring.	Integrated SDB and asthma management.	Specific management strategies to improve asthma control in patients with chronic snoring.
Pollen allergies are common risk factors for SDBs in children [7].	Assessment of the presence of allergic rhinitis in children.	Evaluation of respiratory parameters in sleep.	Pollen count in the management of SDB in children with allergic rhinitis.
In children with allergic rhinitis, an increase in grass pollen count is associated with an increase in SDB severity [7].	Evaluation of pollen count during polysomnography.	Monitoring of pollen-related respiratory parameters.	Consider pollen count as an influential factor on SDB severity in children with allergic rhinitis.
Pollen count should be considered when choosing treatment options for children with allergic rhinitis and SDB [7].	Consideration of pollen count in treatment management.	Personalization of therapies for allergic rhinitis and SDB.	Better control of SDBs in children with allergic rhinitis during pollen exposure.
SDBs are common in allergy practices [8].	Evaluation of nocturnal respiratory symptoms.	Medical therapy for the management of inflammatory conditions of the upper airways.	The proactive role of allergists in the diagnosis and management of SDBs.
There is a bidirectional relationship between asthma and SDBs in children [16].	Assessment of asthma control and SDB Risk.	Appropriate asthma control and allergy therapy.	Asthma control and appropriate use of allergy medications in SDB management.
Asthma and allergic rhinitis can affect sleep and functioning during the day.	Assessment of atopic diseases and their impact on sleep and quality of life [3,10,17].	Appropriate use of medications to reduce nocturnal symptoms and improve sleep.	Management and prevention of SDB in allergic diseases.
The presence of OSA has been associated with asthma exacerbations and reduced quality of life.	PSG for OSA diagnosis; peak expiratory flow measurement (PEFR) for asthma diagnosis [16].	Use of the Pediatric Asthma Quality of Life (PAQLQ) questionnaire to assess quality of life [10].	To consider OSA in asthma management in the hopes of improving quality of life among children with asthma.

Legend: OSA—obstructive sleep apnea; SDB—sleep disordered breathing.

## References

[1] Bitners A.C., Arens R. (2020). Evaluation and Management of Children with Obstructive Sleep Apnea Syndrome. Lung.

[2] Asher M.I., Montefort S., Bjorksten B., Lai C.K., Strachan D.P., Weiland S.K., Williams H. (2006). Worldwide time trends in the prevalence of symptoms of asthma, allergic rhinoconjunctivitis, and eczema in childhood: Isaac phases one and three repeat multicountry cross-sectional surveys. Lancet.

[3] Koinis-Mitchell D., Craig T., Esteban C.A., Klein R.B. (2012). Sleep and allergic disease: A summary of the literature and future directions for research. J. Allergy Clin. Immunol..

[4] Liu J., Wu Y., Wu P., Xu Z., Ni X. (2020). Analysis of the impact of allergic rhinitis on the children with sleep disordered breathing. Int. J. Pediatr. Otorhinolaryngol..

[5] Oka S., Goto T., Hirayama A., Faridi M.K., Camargo C.A., Hasegawa K. (2020). Association of obstructive sleep apnea with severity of patients hospitalized for acute asthma. Ann. Allergy Asthma Immunol..

[6] Gunnlaugsson S., Abul M.H., Wright L., Petty C.R., Permaul P., Gold D.R., Gaffin J.M., Phipatanakul W. (2021). Associations of Snoring and Asthma Morbidity in the School Inner-City Asthma Study. J. Allergy Clin. Immunol. Pract..

[7] Walter L.M., Tamanyan K., Nisbet L., Weichard A.J., Davey M.J., Nixon G.M., Horne R.S.C. (2019). Pollen levels on the day of polysomnography influence sleep disordered breathing severity in children with allergic rhinitis. Sleep Breath..

[8] Shusterman D., Baroody F.M., Craig T., Friedlander S., Nsouli T., Silverman B. (2017). Role of the Allergist-Immunologist and Upper Airway Allergy in Sleep-Disordered Breathing. J. Allergy Clin. Immunol. Pract..

[9] D’Elia C., Gozal D., Bruni O., Goudouris E., Meira E.C.M. (2022). Allergic rhinitis and sleep disorders in children—coexistence and reciprocal interactions. J. Pediatr..

[10] Giraldo-Cadavid L.F., Perdomo-Sanchez K., Córdoba-Gravini J.L., Escamilla M.I., Suarez M., Gelvez N., Gozal D., Duenas-Meza E. (2020). Allergic Rhinitis and OSA in Children Residing at a High Altitude. Chest.

[11] Nyenhuis S.M., Akkoyun E., Liu L., Schatz M., Casale T.B. (2020). Real-World Assessment of Asthma Control and Severity in Children, Adolescents, and Adults with Asthma: Relationships to Care Settings and Comorbidities. J. Allergy Clin. Immunol. Pract..

[12] Sherrey J., Biggs S., Dorrian J., Martin J., Gold M., Kennedy D., Lushington K. (2022). Allergic disease, sleep problems, and psychological distress in children recruited from the general community. Ann. Allergy Asthma Immunol..

[13] Zaffanello M., Ferrante G., Zoccante L., Ciceri M.L., Nosetti L., Tenero L., Piazza M., Piacentini G. (2023). Predictive Power of Oxygen Desaturation Index (ODI) and Apnea-Hypopnea Index (AHI) in Detecting Long-Term Neurocognitive and Psychosocial Outcomes of Sleep-Disordered Breathing in Children: A Questionnaire-Based Study. J. Clin. Med..

[14] Esteban C.A., Everhart R.S., Kopel S.J., Klein R.B., Koinis-Mitchell D. (2017). Allergic sensitization and objective measures of sleep in urban school-aged children with asthma. Ann. Allergy Asthma Immunol..

[15] Zaffanello M., Gasperi E., Tenero L., Piazza M., Pietrobelli A., Sacchetto L., Antoniazzi F., Piacentini G. (2017). Sleep-Disordered Breathing in Children with Recurrent Wheeze/Asthma: A Single Centre Study. Children.

[16] Guo Y., Zhang X., Liu F., Li L., Zhao D., Qian J. (2021). Relationship between Poorly Controlled Asthma and Sleep-Related Breathing Disorders in Children with Asthma: A Two-Center Study. Can. Respir. J..

[17] Trivedi M., El Mallah M., Bailey E., Kremer T., Rhein L.M. (2017). Pediatric Obstructive Sleep Apnea and Asthma: Clinical Implications. Pediatr. Ann..

